# Supplementary material for: End Sequence Analysis Toolkit (ESAT) expands the extractable information from single-cell RNA-seq data
Source: Genome Res. 2016 Oct;26(10):1397–410. doi: 10.1101/gr.207902.116 (PMC5052061; doi:10.1101/gr.207902.116)
Supplement: Supplemental Material [file supp_gr.207902.116_Supp_Materials.tar.gz › ESAT-esat_v0.1/doc/html/classumms_1_1core_1_1coordinatesystem_1_1_transcriptome_space-members.html]

ESAT: Member List


ESAT


- Main Page
- Related Pages
- Packages
- Classes
- Files

- Class List
- Class Index
- Class Hierarchy
- Class Members

All Classes Namespaces Files Functions Variables Enumerator Pages

- umms
- core
- coordinatesystem
- TranscriptomeSpace

umms.core.coordinatesystem.TranscriptomeSpace Member List

This is the complete list of members for umms.core.coordinatesystem.TranscriptomeSpace, including all inherited members.

|  |  |  |
| --- | --- | --- |
| getChromosomeNames() | umms.core.coordinatesystem.TranscriptomeSpace |  |
| getEntireChromosome(String chrName) | umms.core.coordinatesystem.TranscriptomeSpace |  |
| getFragment(String chr, int start, int end) | umms.core.coordinatesystem.TranscriptomeSpace |  |
| getFragment(Annotation annotation) | umms.core.coordinatesystem.TranscriptomeSpace |  |
| getLength() | umms.core.coordinatesystem.TranscriptomeSpace |  |
| getLength(String chr) | umms.core.coordinatesystem.TranscriptomeSpace |  |
| getOverlappingRegion(String chr, int start, int end) | umms.core.coordinatesystem.TranscriptomeSpace |  |
| getPositionCountMap(Gene region, AlignmentModel data) | umms.core.coordinatesystem.TranscriptomeSpace |  |
| getReferenceAnnotation(String name) | umms.core.coordinatesystem.TranscriptomeSpace |  |
| getReferenceAnnotations() | umms.core.coordinatesystem.TranscriptomeSpace |  |
| getReferenceNames() | umms.core.coordinatesystem.TranscriptomeSpace |  |
| getSize(Annotation region) | umms.core.coordinatesystem.TranscriptomeSpace |  |
| getTranscriptomeSpace(String bedFile) | umms.core.coordinatesystem.TranscriptomeSpace | static |
| getWindowIterator(Collection< Gene > baseGenes, int windowSize, int overlap) | umms.core.coordinatesystem.TranscriptomeSpace |  |
| getWindowIterator(int windowSize, String chr, int start, int end, int overlap) | umms.core.coordinatesystem.TranscriptomeSpace |  |
| getWindowIterator(Annotation window, int windowSize, int overlap) | umms.core.coordinatesystem.TranscriptomeSpace |  |
| getWindowIterator(String chr, int windowSize, int overlap) | umms.core.coordinatesystem.TranscriptomeSpace |  |
| getWindowIterator(int windowSize, int overlap) | umms.core.coordinatesystem.TranscriptomeSpace |  |
| hasChromosome(String chr) | umms.core.coordinatesystem.TranscriptomeSpace |  |
| isValidWindow(Annotation a) | umms.core.coordinatesystem.TranscriptomeSpace |  |
| moveAnnotation(Annotation read, int relativeStart, Annotation gene) | umms.core.coordinatesystem.TranscriptomeSpace |  |
| PERMUTATION\_ATTEMPTS | umms.core.coordinatesystem.TranscriptomeSpace | static |
| permuteAnnotation(Annotation a) | umms.core.coordinatesystem.TranscriptomeSpace |  |
| permuteAnnotation(Annotation a, Annotation bounds) | umms.core.coordinatesystem.TranscriptomeSpace |  |
| TranscriptomeSpace(Map< String, Collection< Gene >> chrToGenesMap) | umms.core.coordinatesystem.TranscriptomeSpace |  |
| TranscriptomeSpace(File bamFile, String genomeSeq) | umms.core.coordinatesystem.TranscriptomeSpace |  |
| TranscriptomeSpace(File bamFile, Map< String, Collection< Gene >> chrToGenesMap, String genomeSeq) | umms.core.coordinatesystem.TranscriptomeSpace |  |


---

Generated on Fri Aug 15 2014 15:11:10 for ESAT by  

 1.8.7
